# New Strategy for Inducing Surface Anisotropy in Polyimide Films for Nematics Orientation in Display Applications

**DOI:** 10.3390/nano11113107

**Published:** 2021-11-18

**Authors:** Elena-Luiza Epure, Iuliana Stoica, Raluca Marinica Albu, Camelia Hulubei, Andreea Irina Barzic

**Affiliations:** 1 Faculty of Chemical Engineering & Environmental Protection, Gheorghe Asachi Technical University, 700050 Iasi, Romania; lepure@tuiasi.ro; 2 Petru Poni Institute of Macromolecular Chemistry, 41A Aleea Grigore Ghica Voda, 700487 Iasi, Romania; albu.raluca@icmpp.ro (R.M.A.); hulubei@icmpp.ro (C.H.)

**Keywords:** polyimide, molecular modeling, optical properties, rubbing, morphology, liquid crystals

## Abstract

The operability of liquid crystal displays is strongly impacted by the orientation aspects of nematics, which in turn are affected by the alignment layer surface features. In this work, two polyimide (PI) structures are obtained based on a cycloaliphatic dianhydride and aromatic or aliphatic diamines with distinct flexibility. The attained PI films have high transmittance (T) for visible radiations, i.e., at 550 nm T > 80%. Here, a novel strategy for creating surface anisotropy in the samples that combines rubbing with a cloth and stretching via pressing is reported. Birefringence and atomic force microscopy (AFM) scans reveal that the generated orientation of the chains is affected by the chemical structure of the polymer and order of the steps involved in the surface treatment. Molecular modeling computations and wettability tests show that the PI structure and produced surface topography are competitive factors, which are impacting the intensity of the interactions with the nematic liquid crystals. The achieved results are of great relevance for designing of reliable display devices with improved uniform orientation of liquid crystals.

## 1. Introduction

Transparent polymers can be regarded as the cornerstone for the progress in electronic and optoelectronic industries. Their optical clarity combined with film dimensional stability and flexibility are paramount features for many actual technologies, such as optical lenses, organic light-emitting diodes, optical waveguides, solar cells, and liquid crystal (LC) displays [1]. The latter type of devices has proliferated in a huge number of photonic products used in our daily life, ranging from pocket calculators to TVs and computers [2]. The LC cell is composed of a thin nematic layer comprised between two plates of indium tin oxide (ITO), covered with a transparent polymer film. The gap between the two ITO substrates is small (i.e., a few micrometers). The role of the polymer layer found on top of the transparent conductive oxide is to generate uniform planar alignment of the LC in the vicinity of the surface and, for this reason, this element is called the alignment layer (AL) [3]. The orientation directions of the two AL surfaces are not placed in a parallel manner; instead, they are put at 90° with respect to each other. By following this procedure, the LC director is rotating by 90° within the gap made between the aligning surfaces [2,4]. The working principle of an LC cell is relatively simple and is relying on the capability of the LC molecules to arrange in the presence or absence of an external electric field. At the same time, the quality of the nematic orientation is affected by the morphological characteristics of the AL. The aspects arising from the polymer surface topography could influence elastic strain energy and through this the LC alignment [5]. A common technique for adapting the surface properties of the AL involves unidirectional rubbing with cloth [6]. Fewer reports deal with stretching of the polymer film to generate the desired topography [7,8]. In the case of rubbing, after the textile material contact with the polymer surface, one can observe the appearance of microscopic grooves formed along the rubbing direction [9]. This technique induces local heating and orientation of the chain segments as a result of the deformation caused by rubbing. When casting an LC compound on such textured polymer surfaces, the mesogen acquires a macroscopic alignment. According to Berreman [5], besides the AL surface morphology, there is another factor contributing to the LC orientation, namely, the short-range molecular forces acting among the molecules positioned in a preferential direction. Stöhr and Samant [6] further analyzed the LC alignment mechanism and revealed that the LC orientation direction is determined by an asymmetry in the molecular bonds at the rubbed AL surface. Other important aspects arise from the influence of the rubbing conditions (push length, rotation speed) [10] and the features (size, malleability) of the rubbing cloth fibers [11].

Aside from the surface morphology, for AL uses, the polymer layer must also meet several criteria including thermal stability, good wettability of the nematic, and elevated transparency in the visible domain [2]. Polyimides (PIs) are known to possess such advantageous properties and their structure enables many possibilities for molecular interactions with the LC molecules. For wholly aromatic PIs, the inter/intra-molecular charge transfer complex (CTC) is causing absorption and coloration issues, which are impeding their use as ALs [12]. An alternative solution to this entails the introduction in the PI main chain of flexible bonds, twisting structures and/or bulky substituents (to lower the macromolecule co-planarity), combined with low polarizable atoms or aliphatic moieties [12,13]. Such a PI molecular design approach produces disruption of the CTC interactions and hence renders better solubility and transparency [14,15,16]. In recent works, we showed that the use of cycloaliphatic dianhydrides in the PI synthesis leads to transparent materials, with high glass transition temperature, good dimensional stability, and appropriate adhesion with the LCs [9,17,18,19,20,21]. Moreover, such partially aliphatic PIs have a smooth surface and their morphology can be modified by dynamic plowing lithography [22], rubbing [9,11,23], or by imprinting the banded texture of a lyotropic polymer [24,25].

In this article, two PI films containing cycloaliphatic sequences were prepared and characterized to investigate their suitability as ALs. Molecular modeling computations were performed to evaluate the conformational characteristics of the PIs. There are works in the literature that deal with the development of quantitative structure–property relationship models for different classes of the PIs which predict properties, such as the refractive index, glass transition temperatures, thermal conductivity, dielectric constant, or intrinsic viscosity in polymer–solvent mixtures [26,27,28,29]. Even in our case, with the help of the same method, effective information on the mechanical behavior of the polymers was obtained. The interaction between the liquid crystal molecule and the PI layers was pursued via molecular dynamic simulations.

A novel strategy for the surface patterning was elaborated by alternative rubbing with a cloth and unidirectional stretching and vice versa. The order in which the surface texturing methods are applied is expected to affect the surface features and this is monitored via atomic force microscopy (AFM) measurements. Transmittance and birefringence of the samples are measured for checking their performance in terms of optical clarity and degree of orientation upon surface adaptation. Wettability tests are performed to determine the adhesion at the textured PI films/nematic interface and the results are explained by considering the polymer chemical structure and surface topography. The surface parameters extracted from AFM scans, together with elastic features of the LC, allowed evaluation of the azimuthal anchoring energy of the nematic on the textured samples. All these results bring novel perspectives on the surface design of the ALs for upgrading the LC displays.

## 2. Materials and Methods

### 2.1. Materials and Polyimide Synthesis

The raw materials used for the semi-aliphatic PI preparation: 5-(2,5-dioxotetrahydrol-3-methyl-3- cyclohexene-1,2- dicarboxylic) (N B-4400—denoted here EPI), 4,4′-diaminodiphenyl ether (ODA), and hexamethylenediamine (HMDA) were taken from TCI Chemicals (Paris, France). The solvent 1-methyl-2-pyrrolidinone (anhydrous, 99.5%, NMP) and the nematics N-(4-Methoxybenzylidene)-4-butylaniline (98%, MBBA) and 4′-pentyl-4cyanobiphenyl (98%, 5CB), were purchased from Sigma Aldrich (Taufkirchen, Germany).

**Figure 1:**
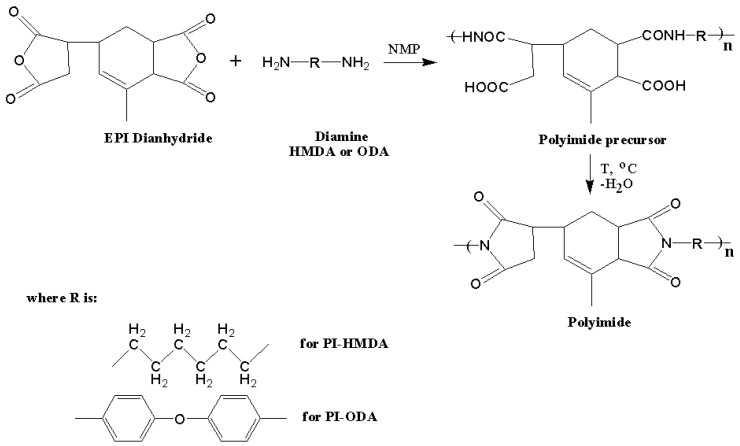
The schematic procedure of synthesis of the EPI-derived polyimides.

The PIs were synthesized using the conventional method of solution polycondensation of the EPI cycloaliphatic dianhydride and HMDA or ODA diamines. Generally, the reaction is undertaken in two steps: the first involves addition of equimolar amounts of monomers in NMP at room temperature and inert atmosphere to form the poly (amic acid) precursor, while the second step requires heating of the system up to ~190 °C to accomplish the cyclodehydration of the precursor and convert it into the corresponding PI structure. Figure 1 illustrates the schematic preparation procedure of the studied PI-HMDA and PI-ODA polymers.

This synthesis procedure is similar to that reported in previous works [30,31]. The studied PI films were attained by pouring the precursor solution on glass substrates and keeping them for 10 h at 80 °C for a slow solvent removal. Then, semi-dried samples were subjected to thermal imidization by heating at 100, 150, 200, and 250 °C (1 h at each stage). In the end, a final treatment was performed for 2 h at 280 °C. The PI films were isolated from the substrate by embedding in hot water, and subsequently, they were dried at 105 °C in vacuum conditions for several hours. The thickness of the films was found to be 60 μm for PI-HMDA and 64 μm for PI-ODA.

### 2.2. Methods

A new method of PI surface modification is proposed, relying on rubbing, followed by stretching and in reverse order from that stated. The rubbing of the samples was achieved with a lab-made device having a rotating cylinder (diameter = 1.2 cm), which moves at 180 rot/min. The material used for surface modification was velvet, which was found on the surface of the cylinder. The rubbing procedure was undertaken for 30 s for each PI film. The film stretching was carried out by subjecting the PI surface to mechanical deformation by pressing with a corrugated metallic surface. During this procedure, the PI films were fixed on a glass substrate (as in LC cell).

All theoretical simulations were performed using commercial Materials Studio 4.0 software [32]. The Synthia module was used for the prediction of the thermophysical and mechanical properties, using the quantitative structure–property relationships (QSPR) method [33]. First, the structural units were shaped and minimized (DMol^3^, PWC functional, 2 × 10^−5^ Ha energy convergence). Afterwards, the polymer chains with 5 repeating units for each structure were minimized as well (Forcite, pcff force field, 2 × 10^−5^ kcal/mol energy convergence). For data reproducibility checking, three amorphous cells for both kinds of polymers were generated, each with a different number of seeds. Every cell has 6 polymeric chains, PI-HMDA, and respectively, PI-ODA. The amorphous cells were built at 298 K, starting from a 0.2 g/cm^3^, reaching a 1.4 g/cm^3^ density. The minimized cells (10^−4^ kcal/mol energy convergence) were subjected to NPT dynamics (conserved amount of substance (N), pressure (P) and temperature (T)) at p = 1 atm, T = 298 K, using a Berendsen thermostat and barostat, for 1 ns. The stable values for the temperature and density indicated the reach to the equilibrium [34]. Another NVT dynamic (conserved amount of substance (N), volume (V), and temperature (T)) for 1 ns provides the elimination of every possible unfavorable interaction from the system.

In a repetitive manner, using the Discover module, NPT dynamics of 100 ps were applied to simulate the stretching of the polymeric film, by Berendsen thermostat and Parrinello barostat [35,36]. Along the X axis of the periodic simulation cell, an external constant stress of 2 GPa was applied. To overcome significant alterations of the cell density, alongside of the Y- and Z-axes a negative stress had to be imposed. Because the main purpose was not to obtain a polymer in a fully stretched state, only 5 iterations were executed. Polymeric surfaces were acquired by sectioning the periodic cells along the xOz plane. Afterwards, the liquid crystal molecules were added. In order to explore the conformational space for searching the lowest energy structures, a quench dynamic was conducted at 550 K or 700 K, NVE ensemble (conserved amount of substance (N), volume (V), and energy (E)). The polymer molecules at the surface were maintained at fixed positions to obtain minimal computational resources [37].

UV-VIS measurements of the solid PI films were performed on a SPECORD 210 PLUS instrument Goebel Instrumentelle Analytik GmbH, Hallertau, Germany).

Birefringence of the modified PI films was determined on a refractometer (DR-M4 model).

Atomic force microscopy measurements were conducted on a NTEGRA Scanning Force Microscope device (NT-MDT Spectrum Instruments, Zelenograd, Russia), with an NSG10 cantilever (with the resonance frequency of 298 kHz), in atmospheric conditions, at 23 °C room temperature, using a semicontact technique in semicontact error SPM mode. The PiezoTube was used as the scanning device with the XY closed-loop on. The set point was around 10 V, the feedback gain 0.4, and the scanning frequency 0.4 Hz. The chosen imaged scan size was 30 × 30 μm^2^. Using Nova v1.1.1.19891 (NT-MDT Spectrum Instruments, Zelenograd, Russia) and Image Analysis 3.5.0.19892 software (NT-MDT Spectrum Instruments, Zelenograd, Russia), the AFM data were acquired and analyzed. The height histograms, bearing curves, and autocorrelation function images obtained from the original topography images, and the 3D texture parameters (Sq—root mean square roughness of the surface, Sdr—surface area ratio, Stdi—surface texture direction index, Sbi—surface bearing index) were calculated from the above-mentioned representations.

Surface wettability of the PI layers in regard to MBBA and 5CB was evaluated by contact angle experiments. Three drops of LC were placed on the film surface to acquire statistical results regarding the interfacial adhesion, with the error being around ±1°.

## 3. Results and Discussion

In this work, two PI samples were tested for AL uses; namely, one that has a fully aliphatic structure (PI-HMDA) and the other with semi-aliphatic structure (PI-ODA). Since these imide-type polymers are synthesized from the same EPI dianhydride, their structural peculiarities are defined by the diamine properties. HMDA has an aliphatic, small, and highly flexible structure, while ODA is an aromatic, bulky, and less flexible molecule. Molecular modeling of the PI structures in the presence/absence of the deformation provides valuable information on the structural aspects that contribute to the induced anisotropy. The morphological, optical, and wettability properties of the ALs are paramount for understanding the performance of the PI layers in terms of anisotropy, which in turn is responsible for the uniform orientation of the LC molecules.

### 3.1. Transmittance and Birefringence of ALs

**Figure 2:**
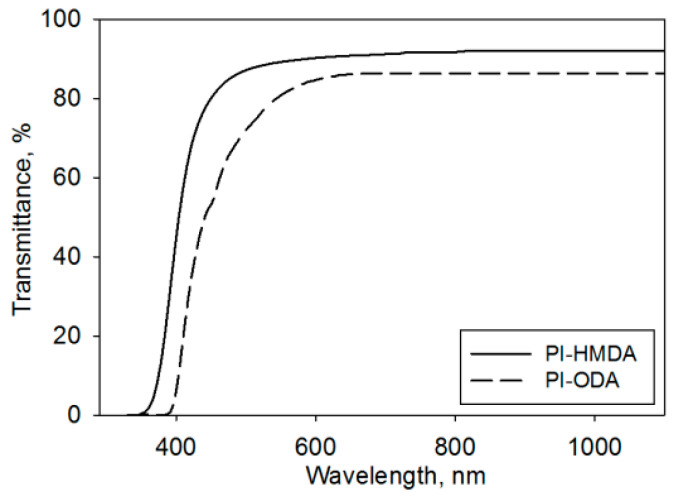
The transmittance data against wavelength recorded for PI-HMDA and PI-ODA free-standing films.

The minimization of the PI absorption in the visible range is highly desirable for AL purposes. PI-HMDA and PI-ODA free-standing films were subjected to spectral analysis. Figure 2 depicts the differences between the transparencies of the fully aliphatic and semi-aliphatic studied PIs. The transmittance (T) measurements are registered from UV to near infrared and the attained data for all samples are indicating good transparency in the visible range, especially for PI-HMDA. For instance, at 500 nm, the polymer containing ODA units presents slightly lower transmittance (T~70%), while the PI derived from HMDA has superior optical properties (T~86%). As the wavelength increases to 700 nm, the transmittance is reaching 91% for PI-HMDA and 86% for PI-ODA. The cut-off wavelength, showing the point where transmittance is below 1%, is 392 nm for PI-ODA and 356 nm for PI-HMDA. The differences in the spectral data are attributed to the distinct features of the diamines. The presence of the aromatic ODA sequences contributes to closer chain packing and CTC interactions among the PI macromolecules, whereas the HMDA renders lower molecular density, smaller polarity, and seldom probability of the CTC occurrence. This is in agreement with the literature, which confirms the contribution of these factors to the transparency of the PIs [12].

The optical band gap energy (E_g_) is a parameter that indicates the energetic threshold required for the light photons to be absorbed. The simplest manner to determine E_g_ relies on its relation to cut-off wavelength:

E_g_ = 1240/λ_c_, (1)

where λ_c_ is the cut-off wavelength, depicting the onset of absorption.

As revealed by Equation (1), the optical band gap is 3.25 eV for PI-ODA and 3.48 eV for PI-HMDA. Hence, for the semi-aliphatic PI, a lower energetic threshold is imposed for allowing the photons’ absorption. At the same time, the fully aliphatic sample has a higher E_g_, which induces a smaller probability of light absorption in this material.

**Figure 3:**
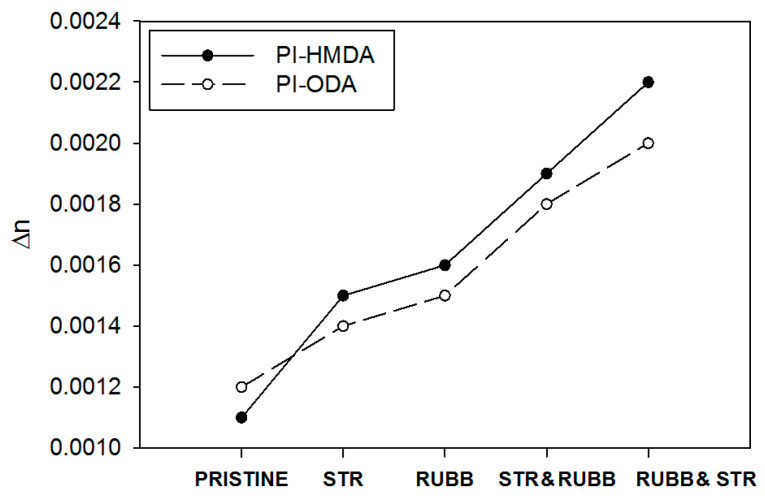
The variation of the birefringence of the pristine and surface adapted PI-HMDA and PI-ODA films.

Birefringence (Δn) is an important optical property for LC display applications. It is widely known as the double refraction of optical radiations in a molecularly ordered material that is expressed by the orientation-dependent differences in the refractive index. This optical parameter contains information on the intrinsic birefringence and orientation distribution function, the latter describing the level of alignment of the molecular units. Thus, birefringence provides an idea about the overall molecular orientation in the samples. Moreover, Δn reveals the distinction among the in-plain and out-of-plain refraction properties, providing data on the 3D structure of the PI, and spatial arrangement of the chain segments that contribute to the light propagation through the layers of the LC cell. As noted in Figure 3, the pristine PI-ODA presents larger birefringence in comparison to PI-HMDA. This is because PIs containing aromatic segments have a higher preference for the macromolecules to orient along the plane of the film. Conversely, when decreasing the aromatic character of the PIs, there is a greater tendency for random orientation of the chains due to weak interactions among them [12,38,39,40]. In other words, the lack of a particular chain’s orientation direction in the film renders low polarizability anisotropy and a reduced birefringence.

Moreover, upon surface modification of the PI films, they gain optical anisotropy, which can be quantified by means of birefringence measurements. According to the data presented in Figure 3, the different Δn values indicate that different degrees of orientation can be generated in the film samples as a function of the applied method of deformation and PI chain flexibility.

The method of stretching by single-step pressing (noted here with STR) is determining the orientation of the macromolecules up to the bulk layers from the PI film. In contrast, during rubbing (noted here with RUBB), the velvet fibers are penetrating less deep in the bulk of the PI film, even if the applied repetitive deformations generate more intense alignment of the chains, which is reflected in a higher anisotropy. When mixing these two procedures, it seems that the first applied technique (RUBB or STR) has the prevalent effect from the point of view of created anisotropy. If the initial method produces higher chain orientation, the second method contributes less to further alignment of the chains. More specifically, when starting with the rubbing step, a larger alignment of the macromolecules is produced, and after stretching, is additionally extended. On the other hand, it can be remarked that as the backbone flexibility is higher, the chains are more sensitive to the applied deformation. Thus, the PI containing ODA units is less able to orient upon the imposed external deformation, while the sample containing flexible HMDA sequences is easier to orient upon the rubbing and/or stretching procedures. Thus, the response to deformation of each studied PI structure has a great impact on the overall birefringence magnitude, explaining the bigger values attained for the modified PI-HMDA films in comparison to PI-ODA.

### 3.2. Molecular Modeling

The first theoretical results were estimated by the Synthia module. The predictive correlations were developed using topological information, based on the connectivity indices derived from graph theory [33]. The most representative thermophysical, mechanical, or entanglement properties at 298 K are shown in Table 1.

Young’s and bulk moduli (the marks of deformation in the elastic region) are representing the level of the solid’s stiffness. If Young’s modulus relates the stress to the straining of a solid along one axis, the bulk modulus reflects its volumetric elasticity. Having the same order of magnitude, the moduli indicate that these are related to each other, and also that the elastic properties are determined by the strength of the inter-chains’ forces [41], especially the van der Waals ones. A high Young’s modulus value for PI-ODA means that this polymer is stiffer than PI-HMDA. The compressibility of a body is mathematically defined as the reciprocal of the bulk modulus. From Table 1, it is noticeable that a great pressure must be applied to compress the PI-ODA polymer, meaning that this polymer is less compressible than the other one. Chain entanglements have a significant impact on the mechanical properties of the polymers [42]. The average length between entanglements of the PI-ODA is less than that of PI-HMDA, showing a high entanglement density in the PI-ODA macromolecular system. This fact could be explained by the large deformations of PI-HMDA than PI-ODA. The strength of the forces that keep the molecules close together in a condensed state, cohesive energy, is well related to the moduli and molecular packing. The theoretical values of the surface tension range similarly to the cohesive forces. This is correct since the strong intermolecular forces mean both high cohesive forces and high surface tensions.

**Figure 4:**
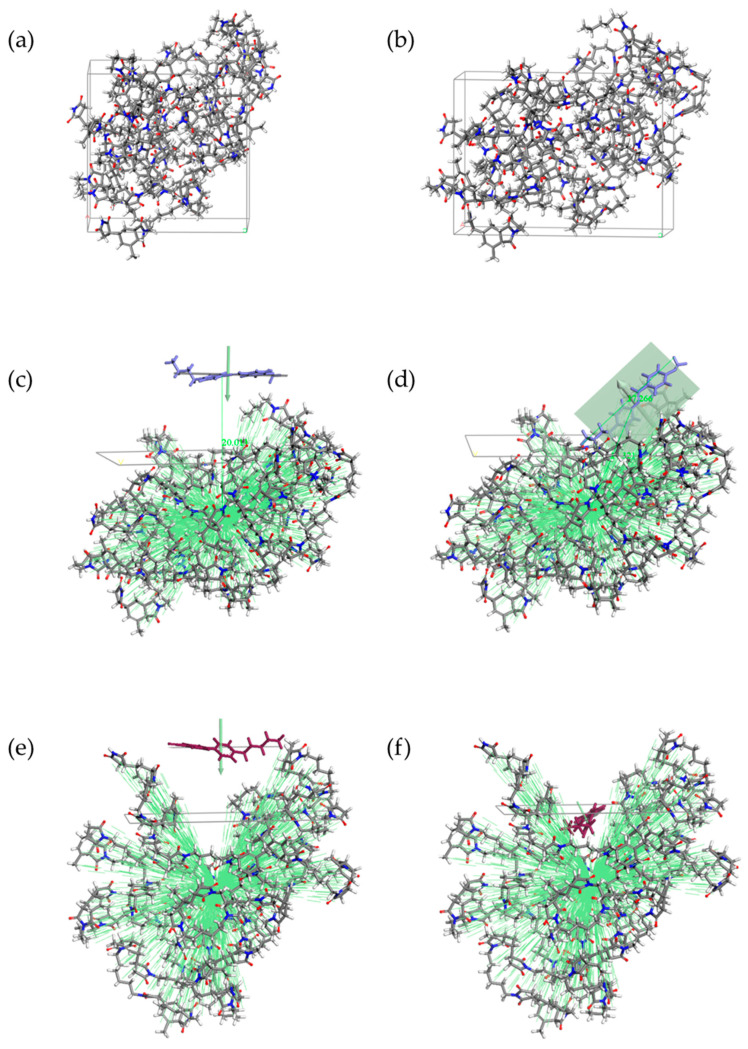
Snapshots of the periodic PI-HMDA cell before (**a**) and after the stretch process (**b**). The polymer layer was limited to 6 polymeric chains. The best fit plane and centroid options were used to obtain similar reference systems with adsorbed MBBA (**c**) and 5CB molecule (**e**). From the multiple outputs of the quench procedure, the system with the lowest energy was chosen for MBBA (**d**), and respectively, for 5CB (**f**).

**Figure 5:**
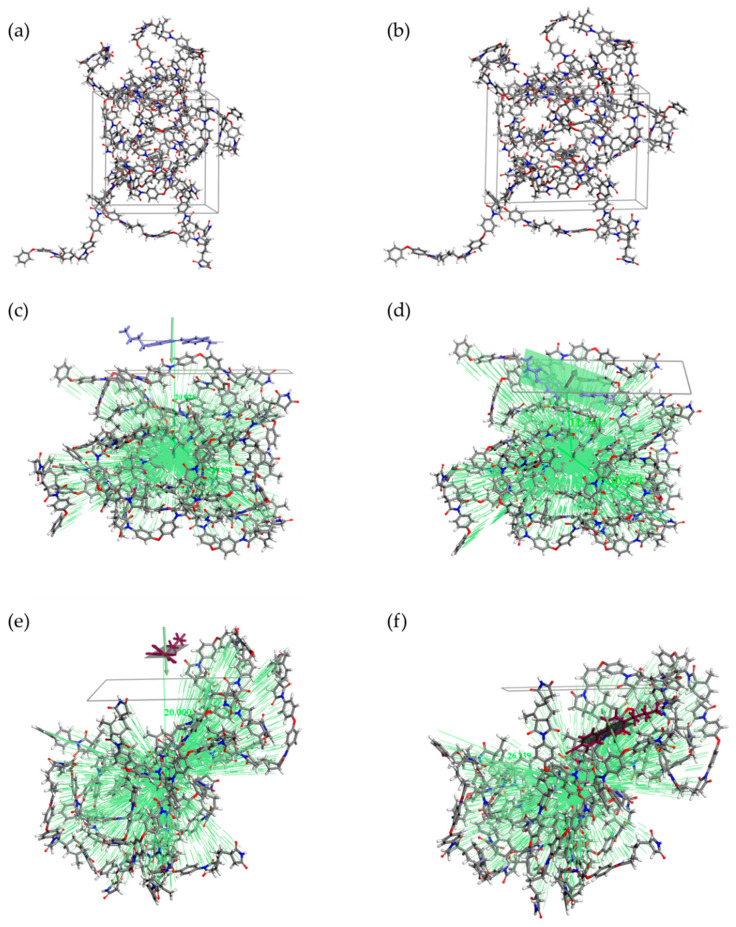
Considering the same procedure and methodology used in Figure 4, here are presented snapshots of the periodic PI-ODA cell before (**a**) and after the stretch process (**b**), and the initial system of the polymer surface and MBBA (**c**), and respectively, 5CB (**e**). From the multiple outputs of the quench procedure, the system with the lowest energy was chosen ((**d**) for system with MBBA, (**f**) for the system with 5CB).

Molecular dynamics simulations were realized for atomic level energetically calculations. After the NPT equilibration of the amorphous cells, the density reaches an average value of 1.13 g/cm^3^ for PI-HMDA (Figure 4) and 1.23 g/cm^3^ for PI-ODA (Figure 5). It was discovered that after the five dynamics, applied in order to stretch the amorphous cell, the elongation along the X axis is only 20%.

For each system, a slight increase in the free volume (for example, PI-HMDA from 36.4% to 37.3%, PI-ODA from 36.7% to 37.1%) was observed. The difference in the packing behavior can be attributed to a subtle growth of the ordering of these systems, obtained through the cell elongation.

As mentioned in the Section 2, the next step was to generate the polymer surface. For a comparative evaluation, the structures with the MBBA or 5CB molecule adsorbed on the polymeric surface were built as follows: (i) a best fit plane of liquid crystal was parallel arranged with the polymeric plane, (ii) the distance from the same N/C atom of the liquid crystal molecule and the centroid of the polymers from the surface was set to 20 Å, (iii) the system was minimized so that the liquid crystal molecule is adsorbed by the polymeric surface, (iv) a quench dynamic, followed by the minimization, provided the most favorable orientation of the liquid crystal molecule on the surface. In order to explore the entire surface, the dynamics for the 5CB systems were conducted at a higher temperature than those used for the MBBA (Figure 4 and Figure 5).

The interaction energy between the polymeric surface and one liquid crystal molecule (MBBA and 5CB, respectively) was calculated in accordance with Equation (2):

ΔE = Epolym/LC − (Epolym + E_LC_) (2)

where Epolym/LC is the total energy of the polymer surface–liquid crystal system, Epolym is single point energy for the system when the liquid crystal molecule is removed_,_ and E_LC_ is the corresponding single point energy for the liquid crystal molecule.

The obtained average values of the interaction energies were: −26.93 kcal/mol for PI-HMDA/MBBA system, −35.70 kcal/mol for PI-ODA/MBBA system, −28.63 kcal/mol for PI-HMDA/5CB system, and −36.85 kcal/mol for PI-ODA/5CB system.

Keeping in mind that a negative value of interaction energy is binding energy, the average of the obtained values indicates that the adsorption strength of the liquid crystal molecule on the PI-ODA surface is higher than the PI-HMDA. Our results suggest that the adhesion of a liquid crystal at the polymeric surface is slightly improved in the PI-ODA case, even though this is a less flexible system than the PI-HMDA. Given the high value of the dipole moment for 5CB (6.16 D), compared to the value obtained for MBBA (1.45 D), it was expected that the binding energy of 5CB would be higher than that of MBBA at the surface of each of the two studied polymers.

Despite the fact that our simplified model may require a series of improvements, it is doubtless a useful tool to investigate the behavior of the liquid crystals at the surface of the examined materials.

### 3.3. Morphological Analysis

**Figure 6:**
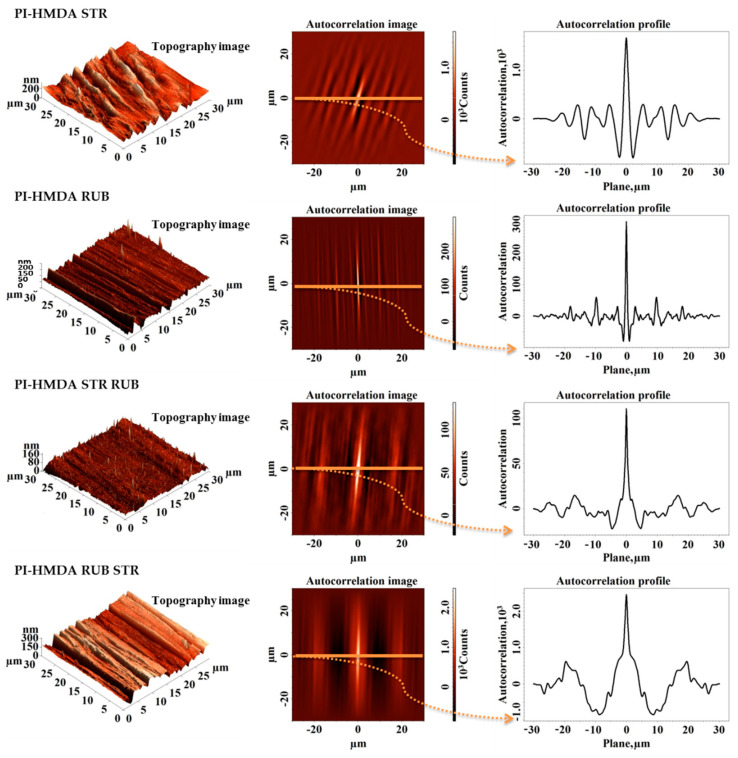
Topography and corresponding autocorrelation images obtained for polyimides PI-HMDA after stretching (STR), rubbing (RUB), rubbing after stretching (STR RUB), and stretching after rubbing (RUB STR).

**Figure 7:**
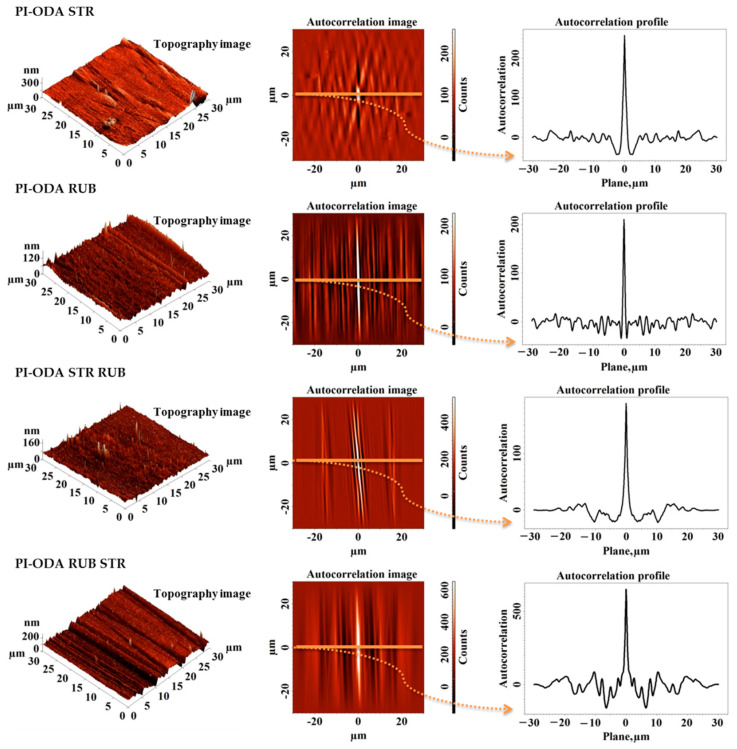
Topography and corresponding autocorrelation images obtained for polyimides PI-ODA after stretching (STR), rubbing (RUB), rubbing after stretching (STR RUB), and stretching after rubbing (RUB STR).

As previously reported in our studies [11,17,18,20,22,23,43], the surfaces of the polyimide samples, whose synthesis is based on EPI, are uniform, homogeneous, presenting the same morphological characteristics in all directions (morphologically isotropic), and with very low roughness, of a few nanometers. Mechanical surface treatments, such as stretching and friction with textiles, can alter these characteristics by inducing the orientation of the morphological features in a certain direction and thus the appearance of the anisotropy (as observed in Figure 6 and Figure 7).

Furthermore, both the order of application of mechanical treatments (stretching—STR, rubbing—RUB, rubbing after stretching—STR RUB, and stretching after rubbing—RUB STR) and the flexibility of the polymer structures are very important factors that can further influence the interactions of the resulted anisotropic surfaces with a nematic liquid crystal.

The determined grooves’ characteristics are listed in Table 2. Analyzing the three-dimensional topographic images acquired for the modified samples (Figure 6 and Figure 7), it can be seen in general that after the stretching process (which induces changes both in bulk and on the surface of the samples) quite deep main grooves are formed at considerable distances from each other (see the grooves’ characteristics: depth of the groove—A and frequency of the grooves—Λ from Table 2). Instead, after the rubbing process (which is mainly a surface process) the formed grooves have shallow depths and the distance between them is much more reduced (Table 2). The prepared films are thicker than 100 nm, which is typical for PI ALs. According to Jiao et al. [4], this could be useful for better control of the voltage. Decreasing PI films’ thickness up to 100 nm would not influence the efficiency of the proposed surface treatment methods, except for the case of stretched PI-HMDA sample, which presents a mean depth of the grooves of 211 nm. Given the finer depth of grooves, generated by the other proposed surface alteration procedures, these approaches are applicable even for PI samples of a standard thickness of 100 nm.

Considering the findings derived from the molecular modeling, according to which PI-ODA is less flexible and less compressible than the PI-HMDA, it can be explained why the oriented morphological structures are much more pronounced in the case of PI-HMDA compared to those obtained for PI-ODA (see the A and Λ values from Table 2). Moreover, the increasing values of the surface feature characteristics favor the enhancement of the root mean square roughness and concomitant the complexity of morphology, suggested by the surface area ratio from Table 2.

The orientation of the polymer molecules was investigated via atomic force microscopy by evaluating the anisotropy of the surface morphology induced by each proposed method of surface treatment (RUBB, STR, RUBB + STR, STR + RUBB). In this way, first it was used as a visual tool, namely, the autocorrelation function (ACF), computed as:

(3) $$\mathrm{ACF}\left(\tau_{x},\;\tau_{y}\right)=\overset{\infty }{\underset{-\infty }{\iint }}z_{1}z_{2}w\left(z_{1},z_{2},\tau_{x},\tau_{y}\right){\mathrm{dz}}_{1}{\mathrm{dz}}_{2}$$

from the surface topography of the AFM image, stored and processed by a computer, in digital form of a matrix z(i, j), where i is the number of points along a scan line and j is the number of lines. In this equation, z_1_ is the value of the height at the point of coordinates (x_1_, y_1_) and z_2_ is the value of the height at the point of coordinates (x_2_, y_2_). Furthermore, the spatial lag along the x scan axis is τ_x_ = x_1_ − x_2_ and the spatial lag along the y scan axis is τ_y_ = y_1_ − y_2_. Corresponding to points (x_1_, y_1_) and (x_2_, y_2_), the function w(z_1_,z_2_,τ_x_,τ_y_) representing a two-dimensional probability density can be defined. This symmetric function, with the maximum value at the origin (zero lag), is susceptible to describe the horizontal spreading of the oriented surface morphologies with repeating features, following the periodicity of the surface and, as the spatial lag increases, asymptotically falling to zero. Usually, in the ACF image the central lobe will be approximately circular (minimum (τ_min_) and maximum (τ_max_) radii being nearly equal) if the surface is morphologically isotropic, presenting no preferential orientation and will be very stretched out (τ_max_ >> τ_min_) if the surface presents a strong privileged orientation, meaning it is morphologically anisotropic.

Besides that, a quantitative tool, namely, the surface texture direction index (Stdi) spatial parameter calculated using the angular power spectral density function (APSDF) applied to the 3D surface was also used to appreciate the orientation of polymer molecules during the surface mechanical processing. As shown in Equation (4),

(4) $$\mathrm{Stdi}=\frac{\sum_{i=0}^{M-1}A\left(\frac{i\pi }{M}\right)}{{\mathrm{MA}}_{\max}}$$

Stdi can be defined as the average amplitude sum divided by the amplitude sum of the major direction, where M is the number of equiangular distinct radial lines and A_max_ is the highest amplitude sum. Being a measure of how dominant the orientation direction is, if the Stdi will be close to values that are towards 0, the surface clearly will be oriented in a dominant direction, being anisotropic, while a Stdi near 1 indicates that the amplitude sum of all directions is similar, and the surface being isotropic, not showing a specific orientation.

In Figure 6 and Figure 7, the autocorrelation images applied as mentioned to the adjacent 3D AFM images, are presented. The data are suggesting a prevalent orientation of the structures and repetitive patterns in spatial space. From X cross-section autocorrelation profiles, taken along the indicated perpendicular direction to the generated grooves, it can be seen that if the spatial conversion increases, autocorrelation asymptotically decreases to zero. Autocorrelation profiles indicate that for the samples processed by a single method, there is only one type of repetitive formation, located at greater distances for stretched samples and smaller for those rubbed with textile material.

**Figure 8:**
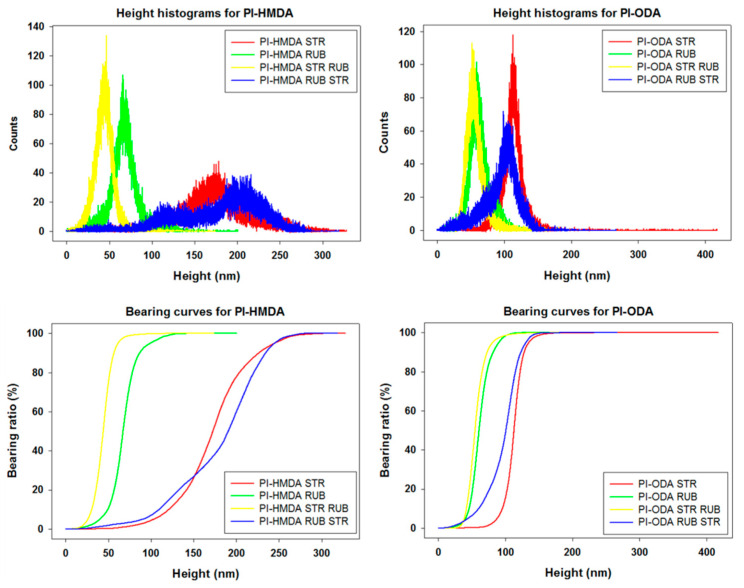
Height histograms and bearing curves obtained from topography images for polyimides PI-HMDA and PI-ODA after stretching (STR), rubbing (RUB), rubbing after stretching (STR RUB), and stretching after rubbing (RUB STR).

When alternating the two methods of surface processing, namely, rubbing after stretching and stretching after rubbing, the autocorrelation images and autocorrelation profiles indicate two types of repetitive formations—the main ones, which are wider, and the secondary ones, which are less wide, located on and between the main ones. It can be seen that the aspect of morphology is predominated by the last applied procedure. There are two phenomena that occur during two-stage processing. When the polyimide films are first stretched, deep grooves are initially formed, which tend to smooth out after rubbing. Conversely, when polyimide films are initially rubbed, they form shallower nanometric grooves, that are much better defined and aligned, which after stretching in the second stage are found on the corrugated relief at the micrometric level. This can easily be seen also from the representations of height distribution (height histogram) and from the bearing area curves (Abbot–Firestone curves), which are presented in Figure 8. The height histograms of the samples processed in a single step have a normal distribution, indicating one single oriented entity. The ones represented for the samples processed in two steps have a bimodal distribution, with two peaks, indicating two orientated entities, as expected. For both PI-HMDA and PI-ODA, the graphs for the stretched samples and rubbed and stretched samples have similar close lower average heights and a narrow aspect. Analogically, the rubbed samples and stretched and rubbed samples present a similar wide aspect and higher average heights. The same trend and proximity are observed in the bearing curves from Figure 8. The above-mentioned thing is reflected in the values of the root mean square roughness and surface aspect ratio from Table 2, which are larger for stretched and rubbed-stretched polymers and lower for the rubbed and stretched-rubbed polymers. The greatest complexity of the morphology (over 0.700%), noticed for the PI-HMDA RUB STR and PI-ODA RUB STR, suggests the increase of the ability for adherent coupling of the nematic liquid crystals. This is also supported by the high values of the surface bearing index (calculated from the bearing area curves) in the case of stretching after rubbing of PI-HMDA and PI-ODA.

Analyzing quantitatively the morphology anisotropy (see Stdi values from Table 2), it was found that this time, the influence of the first mechanical alignment process is predominant: the rubbing process induces a higher anisotropy, while the stretching method a mild one. Moreover, the chemical structure of the PI has a significant influence on this property of the polymer to exhibit variable physical characteristics as a function of the direction of measurement and observation. Due to the aliphatic, small, and highly flexible structure of the HMDA moieties, the induced anisotropy was always higher in the case of PI-HMDA, compared with the one calculated for PI-ODA, containing aromatic, bulky, and less flexible ODA moieties.

### 3.4. Nematic Wettability and Azimuthal Anchoring Energy

Two types of LC were used in this study, namely, MBBA with low polarity and weak negative dielectric anisotropy and 5CB with a terminal cyano group, which renders polarity, the orientation of the dipole moment, and higher dielectric properties [44]. When the nematic molecules of MBBA or 5CB are found in contact with the surface-modified AL films, it is very important to study the interfacial interactions. They are expressed through the work of adhesion (W_adh_), which is defined as follows:

W_adh_ = (1 + cos θ)∙ɣ_LC_, (5)

where θ is the contact angle at the PI/LC interface and ɣ_LC_ is the surface tension of the nematic LC.

**Figure 9:**
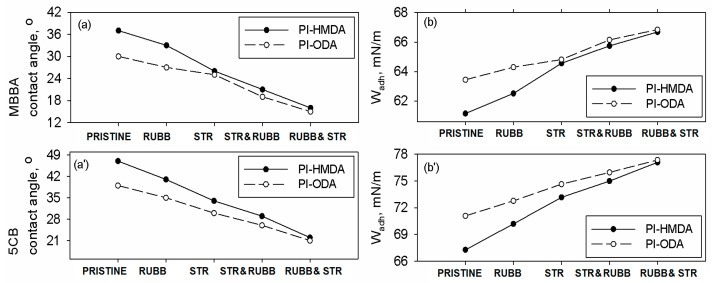
The variation of the (**a**) MBBA and (**a’**) 5CB contact angle and work of adhesion of (**b**) MBBA and (**b’**) 5CB with the surface treatment applied to the PI film.

By measuring the contact angle made by nematic drops on the pristine and surface modified PI films, it is possible to estimate the adhesion at the interface. The contact angles with the LC are measured with the observer orthogonally positioned in regard to the created grooves on the PI surface. In the case of unmodified samples, it seems that the surface polarity is the main factor that dictates the magnitude of the contact angle. Hence, as seen in Figure 9a, pristine PI containing more polar ODA units has a lower value of the MBBA and 5CB contact angle than PI-HMDA.

Upon surface adaptation of the PI foils, the values of θ are affected by the competing effects generated by surface topography and the surface polarity. Since rubbing penetrates in the superficial layers of the sample and induces considerable anisotropy, the value of the contact angle decreases in comparison to the pristine PI films. Similar effects are noted for the stretching procedure, but in the case of PI-ODA, the surface polarity produces a higher decrease of the θ value. When mixing rubbing with stretching, the differences between values of θ for PI-ODA and PI-HMDA become closer. The contact angle values of the MBBA on the samples are slightly slower than those recorded for 5CB (see Figure 9a,a’). All these aspects are reflected in the data acquired for work of adhesion, depicted in Figure 9b. One may remark that the aliphatic (less polar) nature of PI-HMDA maintains slightly lower W_adh_ values than PI-ODA, but the backbone flexibility enables higher anisotropy and a relatively bigger depth of the micro-grooves, which decrease the contact angle. In the case of 5CB, the magnitude of the work of adhesion is higher than that noted for MBBA, regardless of the used surface modification procedure (see Figure 9b,b’).

The alignment of the nematic molecules due to the presence of a solid support is named surface anchoring [45]. The anchoring energy is related to the free energy density of the LC in a system when the director is digressing from the easy axis with a low angle. Thus, the anchoring abilities of the polymer support provide quantitative information on the orienting substrate azimuthal anchoring magnitude generated by the strong interaction of chemically analogous mesogenic parts. According to Berreman [46], the LC anchoring is mainly caused by the elastic distortions produced via contact with a sinusoidally grooved support. A recent approach emphasizes that the principal alignment mechanism of rubbed ALs relies on epitaxial growth of the nematic molecules as a result of macromolecules reorientation [47]. Hence, the surface topographical features are affecting the arrangement of the LC in the LC cell. In contact with the undulated PI surface made of grooves with specific depth and frequency, the azimuthal anchoring energy can be evaluated based on Equation (6):

W_anch_ = 1/4 K_11_ (A q)^2^ q, (6)

where W_anch_ is the azimuthal anchoring energy, A is the depth of the micro-grooves, K_11_ is splay elastic constant of the LC, and q is related to frequency of the grooves Λ = 2 π/q.

Given the fact that the LC is permuted from one layer to the next one, it is presumed that the permutation near the polymer film is impacted by the value of the W_anch_ parameter. Introducing in Equation (6) the surface features of the textured polyimide films, registered in the AFM scans (Table 2), the values of the azimuthal anchoring energy were estimated. As observed in Table 3, the W_anch_ parameter of both MBBA and 5CB ranges significantly with the surface features created either by rubbing, stretching, or their mixed methods. This is because each surface adaptation step induces distinct depths and distances between the micro-grooves. For the stretched or rubbed PI-HMDA films, the azimuthal anchoring energy of MBBA and 5CB values are bigger than those of PI-ODA samples, but upon mixing the methods this changes. Regardless of the used LC, for PI-HMDA foils subjected to stretching followed by rubbing, the level of the W_anch_ is slightly lower than that of the PI-ODA film, whereas for rubbing and stretching this parameter is below the value corresponding to the semi-aliphatic polyimide structure. Thus, when mixing the two surface adaptation techniques, it is noted that the sample with the highest anisotropy requires less energy for digressing the LC from the preferred direction. On the other hand, a larger surface anchoring energy of the PI-ODA reveals a better interaction with the LC molecules, probably also to its higher surface polarity. Regardless of the PI structure or surface modification method, the magnitude of the azimuthal anchoring energy is higher for 5CB nematic than that attained for MBBA. If the AL surface has large azimuthal anchoring energy, this is favoring homogeneous alignment of LCs, which could be employed to store some information. Conversely, if the main purpose is to attain improved characteristics in dynamic processes, such as the low-voltage switching of LCs from planar (or tilted) to homeotropic orientation (vertical), it is highly desirable to make ALs with weak azimuthal anchoring energy [48]. Based on the latter issue, it can be noted that the fully aliphatic sample displays the best features for use as AL in LC displays.

## 4. Conclusions

Two PI structures based on the same cycloaliphatic dianhydride were synthesized and characterized to check their suitability as ALs. The polymer films are highly transparent, i.e., at 550 nm T > 80%. Upon surface modification, the PI foils gain anisotropy, which correlated through birefringence revealed higher chain orientation for the PI-HMDA sample in comparison to PI-ODA.

The mechanical behavior of both polymers was quantitatively predicted. In order to understand the competition between adsorbed liquid crystal molecules on the polymeric surfaces, molecular dynamics simulations were conducted. It can be noted that even the PI-ODA is less flexible and compressible than PI-HMDA, the stretched PI-ODA film has the most favorable interactions with the LC molecules, this fact being emphasized in an experimental manner later. Both theoretically and experimentally, it has been found that 5CB molecules have better adhesion to polymeric surfaces.

AFM measurements revealed that the polymer structure and the order of application of the mechanical treatments are key factors that can further influence the interactions of the resulted anisotropic surfaces with a nematic liquid crystal. The oriented morphological structures were much more pronounced and had higher anisotropy in the case of softer and easily compressible PI-HMDA compared to those obtained for the stiffer and less compressible PI-ODA. The induced micro and nano-grooves aspect, correlated with the roughness, complexity, and bearing properties was predominated by the last applied process, either stretching or rubbing. Instead, the morphology anisotropy was influenced by the first applied mechanical alignment process: the rubbing process induces a higher anisotropy, while the stretching method a mild one.

The bigger surface polarity of PI-ODA favors better interaction with LC, while the larger anisotropy of PI-HMDA imposes less energy for diverging the LC molecules from their preferred direction of orientation. The magnitude of the azimuthal anchoring energy was higher for 5CB nematic than that attained for MBBA. Given the highest transparency, better surface anisotropy, and lower azimuthal anchoring energy of the fully aliphatic sample, it can be concluded that PI-HMDA has the most suitable features for use as AL in LC displays.

## Figures and Tables

**Table 1:** Predicted parameters of the PI-HMDA and PI-ODA polymers by QSPR method.

System	Bulk Modulus (MPa)	Young’s Modulus (MPa)	Brittle Fracture Stress * (MPa)	Entanglement Length (Å)	Cohesive Energy (Fedors) (kJ/mol)	Surface Tension (Fedors) (dyn/cm)	Density (g/cm^3^)
PI-HMDA	4554.03	2528.81	128.00	200.86	147.14	48.09	1.20
PI-ODA	5297.00	3101.27	117.86	166.69	181.07	49.94	1.28

* At considered molecular weight, 10,000 amu.

**Table 2:** Groove characteristics and 3D texture parameters obtained for polyimides PI-HMDA and PI-ODA after stretching (STR), rubbing (RUB), rubbing after stretching (STR RUB), and stretching after rubbing (RUB STR).

Sample	Groove Characteristics		3D Texture Parameters			
	A	Λ	Sq	Sdr	Stdi	Sbi
PI-HMDA STR	211 ± 24	3686 ± 555	41	0.556	0.298	0.497
PI-HMDA RUB	39 ± 16	706 ± 129	17	0.424	0.137	0.170
PI-HMDA STR RUB	22 ± 5	953 ± 256	11	0.286	0.385	0.102
PI-HMDA RUB STR	74 ± 15	1422 ± 567	50	0.700	0.157	0.670
PI-ODA STR	54 ± 12	1923 ± 297	16	0.382	0.480	0.0568
PI-ODA RUB	20 ± 6	657 ± 191	15	0.328	0.214	0.168
PI-ODA STR RUB	33 ± 15	1026 ± 190	14	0.374	0.448	0.124
PI-ODA RUB STR	65 ± 17	1112 ± 142	25	0.737	0.179	0.187

A: depth of the groove (nm); Λ: frequency of the grooves (nm); Sq: root mean square roughness of the surface (nm); Sdr: surface area ratio (%); Stdi: surface texture direction index; Sbi: surface bearing index.

**Table 3:** The values of the azimuthal anchoring energy of MBBA and 5CB on the polyimides PI-HMDA and PI-ODA after stretching (STR), rubbing (RUB), rubbing after stretching (STR RUB), and stretching after rubbing (RUB STR).

Sample	Azimuthal Anchoring Energy, N/nm	
	MBBA	5CB
PI-HMDA STR	3.1926 × 10^−16^	3.7431 × 10^−16^
PI-HMDA RUB	1.5523 × 10^−15^	1.8199 × 10^−15^
PI-HMDA STR RUB	2.0082 × 10^−16^	2.3545 × 10^−16^
PI-HMDA RUB STR	6.8393 × 10^−16^	8.0185 × 10^−16^
PI-ODA STR	1.4726 × 10^−16^	1.7265 × 10^−16^
PI-ODA RUB	5.0654 × 10^−16^	5.9387 × 10^−16^
PI-ODA STR RUB	2.1223 × 10^−16^	2.4882 × 10^−16^
PI-ODA RUB STR	1.1035 × 10^−15^	1.2937 × 10^−15^
